# Repairing Instruments

**Published:** 1848-01

**Authors:** 


### REPAIRING INSTRUMENTS.

Dental instruments are rarely ever obtained from the Cutler, fully fitted for the purposes intended. The points or edges of all, from scaling instruments to forceps, must have the impress of the owner's mechanical ability and judgment. Excavators, plugging instruments and drills, often require to be suddenly re-pointed and tempered, and not unfrequently, entire new ones are to be made. These duties, every Dental Surgeon should be prepared to perform with neatness aud dispatch, and for that purpose, a spirit-lamp, a small blow pipe, some files, which are expressly kept for the purpose, a pair of round and flat plyers, some fine emery or other polishing material, with buffs of hard wood, a little water for tempering, in a suitable vessel, and an Arkansas oil stone, should always be in readiness, whenever the dentist is forced to become his own instrument maker.

Files for the laboratory and operating drawer, possessing every variety of shape and curve, may be conveniently formed, by heating old or new files red hot, and bending them while so, to any required shape. The points and shanks may then be filed to suit the convenience of the operator, while the temper can be immediately restored, by heating them to a temperature between a red and white heat, and plunging them into cold water. A charcoal fire should always be used.

A variety of files thus prepared, will be found indespensable to the practitioner who expects to advance, or excel, in the mechanical department of his profession. We trust our brethren will pardon us for what might appear an effort on our part, to force upon them what is not new. We disclaim any originality in the practical suggestions here thrown out, our sole object being to impress upon the minds of the younger members of the profession, the facility, if industrious, with which they may, at all times, be well supplied with every variety of instrument, that circumstances may call upon them to use.—St. L. Ed.

